# Nose-Ology

**Published:** 1859-09

**Authors:** 


### NOSE-OLOGY.

Some persons have an ugly habit of jerking out the little hairs growing inside the nostrils ; the surface from which they grow is exceedingly sensitive, and the slightest touch of one of them causes an itching or a tittilation, which is quite sure to arrest the attention, and thus an effectual guard is placed against insects and worms crawling in during sleep.

In addition, each individual hair resist the passage of air, and altogether they make a valuable respiratior by detaining the very cold air from rushing into the lungs, the fruitful cause of deadly pneumonias (inflammation of the lungs.) And more than all, being so near the gristle, the skin has very little vitality, very little power of healing, and if this healing is baffled at too short intervals by the tearing away of these hairs, that power is soon lost, and a cancerous sore is the result.

Some persons are deluded into the belief that drawing water up through the nose to wash it out is beneficial ; it can only result in clearing off that bland fluid which nature throws out for the lubrication of the parts, and to prevent their becoming dry by the constant passage of the air over them ; all are familiar with that uncomfortable dryness in a common cold. The purest water has great harshness compared with the soft fluids which nature manufactures for her own purposes.

Bleeding from the nose, when spontaneous, should in almost all cases be let alone. It is an effort of nature to relieve herself of internal congestions, of a surplus of blood, often giving instantaneous and grateful relief from headache and other ailments. A teaspoonful of blood from the nose has prevented many a fatal attack of apoplexy ; hence a nose bleeding is sometimes the safety valve of life.

We once saw an infant apparently dying from an over-dose of paregoric, given by an ignorant mother to keep it quiet while travelling in a stage-coach, but by the gushing of blood from the nose, it at once revived and was saved.

It is time enough to interfere with a bleeding from the nose when a tablespoonful has dropped, or when it is seen to come out in a continous stream ; then the patient should sit upright, and have cold water poured on the head, or a cushion of fine ice kept over the whole scalp ; if more is needed, snuff up powdered alum, or alum-water, or the fine dust from a tea-canister, or the scrapings of the inside of tanned leather. A spontaneous bleeding at the nose is nature declaring that there is too much blood in the body ; then, not an atom of food should be eaten for twenty-four hours.

